# Systematic pathway engineering of *Escherichia coli* for enhanced 3’-sialyllactose production

**DOI:** 10.1186/s40643-026-01110-8

**Published:** 2026-08-10

**Authors:** Yan Wu, Taoling Min, Zhenfeng Ma, Shu Wang, Chuanmin Sun, Hanzhi Zhang, Chenxi Huang, Xudong Qu, Zhenhua Tian

**Affiliations:** 1 Abiochem Biotechnology (Group) Co., Ltd, Shanghai, 200241 China; 2 Synaura Biotechnology (Shanghai) Co., Ltd, Shanghai, 200120 China; 3 State Key Laboratory of Microbial Metabolism, School of Life Sciences and Biotechnology, Shanghai Jiao Tong University, Shanghai, 200240 China

**Keywords:** Metabolic engineering, Promoter optimization, Multicopy integration, *α*-2,3-sialyltransferase, Human milk oligosaccharides

## Abstract

3’-Sialyllactose (3’-SL), the simplest sialylated human milk oligosaccharide (HMO), is widely incorporated into infant formula due to its roles in shaping the gut microbiota, inhibiting pathogen adhesion, and supporting neurodevelopment. In this study, we developed an efficient microbial cell factory for 3’-SL production by enhancing carbon utilization and CTP regeneration. Following the identification of a highly active *α*-2,3-sialyltransferase, metabolic flux was redirected through *pfkA* deletion and *fbaA* overexpression. The precursor pool was strengthened by optimizing the expression of key enzymes involved in UDP-GlcNAc and CMP-Neu5Ac biosynthesis. To further improve pathway balance and stability, promoter engineering was applied to optimize the expression of four key genes (*neuA*, *neuB*, *neuC* and *ST*). The optimized multigene cassette was subsequently integrated into the chromosome using a one-step multicopy integration strategy, generating a plasmid-free and antibiotic marker-free production strain. The resulting strain achieved 3’-SL titers of 7.75 g L^−1^ in shake flasks and 102.18 g L^−1^ in a 5-L fed-batch bioreactor, representing, to the best of our knowledge, the highest reported level to date. This work demonstrates a robust strategy combining metabolic rewiring, adaptive gene expression tuning and multicopy genome integration, providing a versatile platform for the sustainable production of 3’-SL and other high-value HMOs.

**Graphical abstract:**

**Figure:**
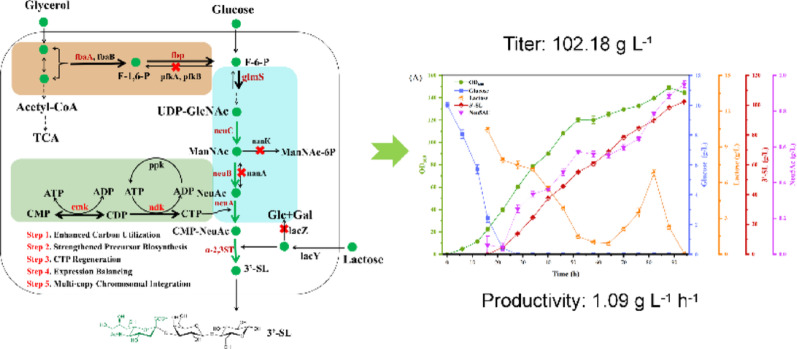

**Supplementary Information:**

The online version contains supplementary material available at 10.1186/s40643-026-01110-8.

## Introduction

Human milk oligosaccharides (HMOs) are the third most abundant solid component in human milk after lactose and lipids, comprising ~ 20% of the total carbohydrates (Bych et al. 2019; Chen et al. 2023). Over 200 structurally distinct HMOs have been identified and can be classified into neutral fucosylated, neutral non-fucosylated, or sialylated (Zhu et al. 2022; Han et al. 2012). Sialylated HMOs account for ~ 13% of the total, with 3’-sialyllactose (3’-SL) and 6’-sialyllactose (6’-SL) being the most abundant. They consist of lactose with Neu5Ac linked through α-2,3 or α-2,6 glycosidic bonds, respectively (Thurl et al. 2017). 3’-SL is of particular interest due to its ability to inhibit pathogen adhesion, reduce necrotizing enterocolitis, modulate immunity, and support neurodevelopment (Lyons et al. 2020; Craft et al. 2019; Lis-Kuberka and Orczyk-Pawiłowicz 2019). Although cow and goat milk contain oligosaccharides, their concentrations (< 1%) and structures differ significantly from human HMOs, rendering them unsuitable as sources (Van Leeuwen et al. 2020; Jiang et al. 2024). With the growing demand for functional infant formula, scalable 3’-SL production is essential. Extraction from milk is impractical, and chemical synthesis is complex and expensive. Microbial fermentation has thus emerged as the most promising route (Fang et al. 2025; Tian et al. 2019).

**Fig. 1:**
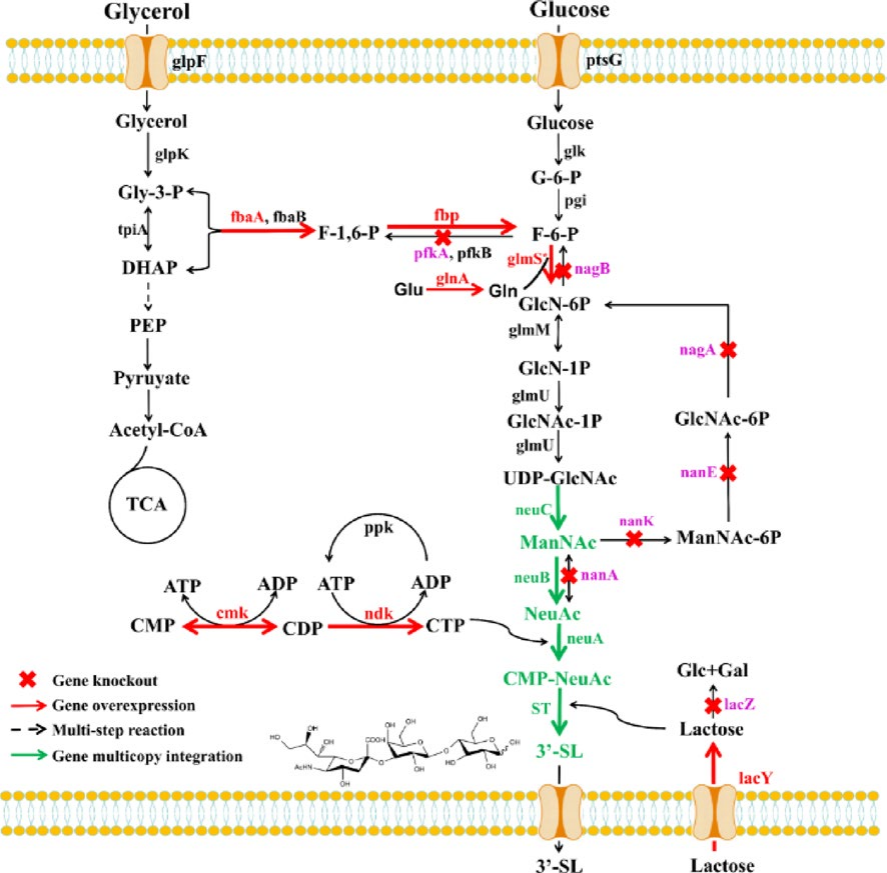
Metabolic pathway for the biosynthesis of 3′-SL in engineered *E. coli* BL21 (DE3). Overexpressed genes are highlighted in red, knockout genes are annotated in purple; multicopy integrate genes are indicated in green; black dotted lines represented multistep reaction. Key gene abbreviations: *glmS*, encoding glucosamine-6-phosphate synthase; *glmM*, encoding glucosamine synthase; *glmU*, encoding UDP-N-acetylglucosamine pyrophosphorylase; *neuC*, encoding UDP-GlcNAc 2-epimerase; *neuB*, encoding Neu5Ac synthase; *neuA*, CMP-Neu5Ac synthetase; *ST*, encoding α2,3-SiaT; *lacZ*, encoding β-galactosidase; *lacY*, lactose permease; *glnA*, glutamine synthetase; *pfkA*, 6-phosphofructokinase; *fbp*, fructose-1,6-bisphosphatase; *fbaA*, fructose-bisphosphate aldolase; *nanA*, encoding Neu5Ac aldolase; *nanK*, encoding ManNAc kinase; *nanE*, encoding N-acetylmannosamine-6-P epimerase; *nagA*, N-acetylglucosamine-6-phosphate deacetylase; *nagB*, glucosamine-6-phosphate deaminase; *cmk*, cytidine monophosphate kinase; *ndk*, nucleoside diphosphate kinase; *ppk*, polyphosphate kinase; Gly-3-P, glyceraldehyde-3-P; DHAP, dihydroxyacetone phosphate; F-1,6-P, fructose-1,6-bisphosphatase; F-6-P, fructose-6-phosphate; GlcN-6-P, glucosamine- 6-phosphate; GlcN-1-P, glucosamine-1-phosphate; UDP-GlcNAc, uridine-5′-diphospho-N-acetylglucosamine; ManNAc, N-acetylmannosamine; Neu5Ac, N-acetylneuraminic acid; CMP-Neu5Ac, cytidine-5′-monophospho-N-acetylneuraminic acid; 3′-SL, 3′-sialyllactose

Recent advances demonstrate the considerable potential of engineered *Escherichia coli* for 3’-SL production. Zhang et al. achieved a 3’-SL titer of 78 g L^−1^ via whole-cell biocatalysis in *E. coli* JM109(DE3) using a yeast-derived CTP regeneration system and exogenous GlcNAc. However, the relatively low molar conversion efficiency (~ 31%) and the requirement for GlcNAc supplementation increased the overall production cost (Zhang et al. 2025). In contrast, de novo biosynthetic routes have attracted increasing industrial and scientific interest because they utilize inexpensive carbon sources, such as glycerol and glucose, while eliminating the need for exogenous GlcNAc. As a result, de novo biosynthesis has emerged as the dominant approach for microbial production of sialylated HMOs. Among these studies, Lv et al. engineered *E. coli* BL21star(DE3)Δ*lacZ* for de novo 3’-SL synthesis from glycerol, reaching 56.8 g L^−1^ in a 5-L bioreactor by optimizing α-2,3-sialyltransferase (ST) expression, disrupting Neu5Ac-consuming pathways, and enhancing CTP recycling (Lv et al. 2024). Another representative study by Li et al. employed a modular three-plasmid system comprising UDP-GlcNAc synthesis (*glmS*, *glmM*, *glmU*), CMP-Neu5Ac synthesis (*neuC*, *neuB*, *neuA*), and *ST*/*lacY* modules (Fig. 1), together with RBS and protein-tag engineering, resulting in a 3’-SL titer of 44.2 g L^−1^ after 84 h of fermentation (Li et al. , 2024b). These studies generally relied on multicopy plasmid-based expression of key pathway genes together with fine-tuning of nucleotide sugar precursor pathways. Collectively, they demonstrate that microbial fermentation is a promising platform for industrial 3’-SL production because of its low substrate cost, operational simplicity, and high production potential.

Despite these advances, most reported de novo 3’-SL biosynthetic systems still rely on multi-plasmid, multicopy expression systems to achieve high pathway activity. Such strategies are often associated with metabolic burden, limited flexibility in balancing pathway expression, genetic instability during prolonged cultivation or scale-up, and dependence on antibiotic selection markers, making them less suitable for food-grade and industrial applications (Zhang et al. 2022; Yue et al. 2025). Furthermore, accumulating evidence indicates that high-level 3’-SL production requires not only efficient precursor supply but also balanced pathway expression and robust genetic architecture. Therefore, developing integrated metabolic engineering strategies that combine carbon utilization, CTP regeneration, and stable chromosomal integration remains essential for improving fermentation performance and industrial applicability.

To address these challenges, we employed the high-Neu5Ac-producing strain SLEB01 as the parental chassis for 3’-SL biosynthesis. A highly active α2,3-sialyltransferase was first identified, followed by systematic engineering of carbon flux, precursor supply, and CTP regeneration to enhance 3’-SL biosynthetic efficiency. To further balance pathway expression, heterologous genes were coordinately regulated using promoters of graded strengths within a multigene expression cassette. Finally, the optimized biosynthetic pathway was chromosomally integrated into *E. coli* BL21(DE3), generating a plasmid-free and antibiotic-free production strain with enhanced genetic stability and efficient 3’-SL production.

## Materials and methods

### Strains, plasmids, and genes

All strains used in this study are listed in Table 1, and all plasmids are summarized in Supporting Table S1. *E. coli* DH5*α* was used for cloning and plasmid construction, whereas SLEB01 served as the host strain for 3’-SL production. Plasmids pCDFDuet-1 and pET28a were used for heterologous protein expression; pCas and pTargetF were employed for gene knock-in; pTU1-A ~ D-RFP and pTU2-A-RFP for assembly of expression modules; and pTet-tns, pTetQCas-IS1, pRE57, and pCutamp for multicopy gene integration.

**Table 1:** Strains used in this study

Strains	Characteristics	Source
BL21(DE3)	F^−^ompT hsdSB(rB^−^mB^−^)gal dcm rnel31λ༈DE3༉	Invitrogen
SLIS026	*BL21(DE3)::ΔlacZ*,* ΔnanA*,* ΔnanKE*	Lab stock
SLEB01	*BL21(DE3)::ΔLacZ ΔnanA ΔnanKE ΔnagABE*	Lab stock
SLEB02	*BL21(DE3)::ΔLacZ ΔnanA ΔnanKE ΔnagABE ΔpfkA*	This study
SLEB03	*BL21(DE3)::ΔLacZ ΔnanA ΔnanKE ΔnagABE ΔmelB::LacY*	This study
SLEB04	*BL21(DE3)::ΔLacZ ΔnanA ΔnanKE ΔnagABE ΔpfkA::LacY*	This study
SLEB05	*BL21(DE3)::ΔLacZ ΔnanA ΔnanKE ΔnagABE ΔpfkA::LacY ΔaslA::fbaA-fbp*	
SLEB06	*BL21(DE3)::ΔLacZ ΔnanA ΔnanKE ΔnagABE ΔpfkA::LacY ΔaslA::fbaA-fbp ΔgltB::glmS-glnA*	This study
SLEB07	*BL21(DE3)::ΔLacZ ΔnanA ΔnanKE ΔnagABE ΔpfkA::LacY ΔaslA::fbaA-fbp ΔgltB::glmM*	This study
SLEB08	*BL21(DE3)::ΔLacZ ΔnanA ΔnanKE ΔnagABE ΔpfkA::LacY ΔaslA::fbaA-fbp ΔgltB::glmU*	This study
SLEB09	*BL21(DE3)::ΔLacZ ΔnanA ΔnanKE ΔnagABE ΔpfkA::LacY ΔaslA::fbaA-fbp ΔgltB::glmS-glnA ΔmelB::CMK-PPK*	This study
SLEB10	*BL21(DE3)::ΔLacZ ΔnanA ΔnanKE ΔnagABE ΔpfkA::LacY ΔaslA::fbaA-fbp ΔgltB::glmS-glnA ΔmelB::NDK-PPK*	This study
S01	SLEB01 carrying plasmids pSL01 and pET28a-*NmST*	This study
S02	SLEB01 carrying plasmids pSL01 and pET28a-*PsST*	This study
S03	SLEB01 carrying plasmids pSL01 and pET28a-*PmST1*	This study
S04	SLEB01 carrying plasmids pSL01 and pET28a-*PmST2*	This study
S05	SLEB01 carrying plasmids pSL01 and pET28a-*BtST*	This study
S06	SLEB01 carrying plasmids pSL01 and pET28a-*VsST*	This study
S07	SLEB01 carrying plasmids pSL01 and pET28a-*NgST*	This study
S08	SLEB01 carrying plasmids pSL01 and pET28a-*PpST*	This study
S09	SLEB02 carrying plasmids pSL01 and pET28a-*BtST*	This study
S10	SLEB03 carrying plasmids pSL01 and pET28a-*BtST*	This study
S11	SLEB04 carrying plasmids pSL01 and pET28a-*BtST*	This study
S12	SLEB05 carrying plasmids pSL01 and pET28a-*BtST*	This study
S13	SLEB06 carrying plasmids pSL01 and pET28a-*BtST*	This study
S14	SLEB07 carrying plasmids pSL01 and pET28a-*BtST*	This study
S15	SLEB08 carrying plasmids pSL01 and pET28a-*BtST*	This study
S16	SLEB09 carrying plasmids pSL01 and pET28a-*BtST*	This study
S17	SLEB010 carrying plasmids pSL01and pET28a-*BtST*	This study
S18	SLEB09 carrying plasmids pSL20	This study
S19	SLEB09 inserting IS1::*neuA*-*neuB*-*neuC*-*BtST* gene cassette, with tool plasmids	This study
S20	SLEB09 inserting IS1::*neuA*-*neuB*-*neuC*-*BtST* gene cassette, without tool plasmids	This study

- Unless stated otherwise, plasmids were constructed using Gibson assembly with the NEBuilder HiFi DNA Assembly Master Mix. Codon-optimized genes encoding UDP-N-acetylglucosamine epimerase *neuC* (WP_002874240.1) and N-acetylneuraminic acid synthase *neuB* (WP_002874241.1) from *Campylobacter jejuni*, CMP-Neu5Ac synthase *neuA* (WP_002215295.1) from *Neisseria meningitidis*, *BtST* (AGH37861.1) from *Bibersteinia trehalosi*, and additional ST variants for screening were synthesized by Beijing Tsingke Biotech Co., Ltd.

### Reagents, media and culture conditions

A 3’-SL standard (≥ 95% purity) was purchased from Carbosynth (Berkshire, UK). Lactose, IPTG, antibiotics (ampicillin, kanamycin, apramycin, chloramphenicol, spectinomycin, tetracycline), and other analytical-grade reagents were obtained from Sangon Biotech (Shanghai) Co., Ltd.

Unless otherwise specified, all concentrations described below refer to final concentrations. The engineered strain for 3’-SL production was initially grown in LB medium (10 g L^−1^ tryptone, 5 g L^−1^ yeast extract, 10 g L^−1^ NaCl) at 37 °C and 250 rpm for 4–5 h. The seed culture (2.5-5% v/v) was inoculated into 100 mL TB medium (10 g L^−1^ tryptone, 18 g L^−1^ yeast extract, 2.31 g L^−1^ KH_2_PO_4_, 16.43 g L^−1^ K_2_HPO_4_, 4 mL L^−1^ glycerol) in a 500 mL flask and cultured under the same conditions until OD_600_ reached 1.0-1.2. Fermentation was then initiated by adding 0.1 mM IPTG, 2 g L^− 1^ MgSO_4_·7 H_2_O, 0.1 mL trace-element solution (54.4 g L^−1^ (NH_4_)_2_Fe(C_6_H_5_O_7_)_2_, 9.8 g L^−1^ MnCl_2_·4 H_2_O, 1.6 g L^−1^ CoCl_2_·6 H_2_O, 1 g L^−1^ CuCl_2_·2 H_2_O, 1.9 g L^−1^ H_2_BO_2_, 9 g L^−1^ ZnSO_4_·7 H_2_O, 1.1 g L^−1^ Na₂MoO_4_·2 H_2_O, 1.5 g L^−1^ Na_2_SeO_2_, 1.5 g L^−1^ NiSO_4_·6 H_2_O), together with 10 g L^−1^ glucose, and 5 g L^−1^ lactose, followed by incubation at 30 °C and 250 rpm. Glucose (10 g L^−1^) was supplemented every 12 h, and fermentation was terminated at 48 h. For plasmid-bearing strains, antibiotics were added at the following concentrations: ampicillin 100 µg mL^−1^, kanamycin 50 µg mL^−1^, streptomycin 50 µg mL^−1^, apramycin 50 µg mL^−1^, chloramphenicol 25 µg mL^−1^, and tetracycline 100–1000 ng mL^−1^.

### Gene editing

Chromosomal gene knockouts in *E. coli* BL21(DE3) were performed using the CRISPR–Cas9 system consisting of plasmid pCas9 (expressing Cas9 nuclease) and pTargetF (carrying sgRNA with a target-specific N20 sequence). sgRNAs were designed using the CRISPOR online tool (https://crispor.gi.ucsc.edu/). Gene editing was performed as previously described (Li et al. 2021a). Plasmid information is provided in Supporting Table S1, and primers are listed in Supporting Table S2.

### Balance of key enzymes expression in 3’-SL synthesis

To optimize expression compatibility of *neuA*, *neuB*, *neuC*, and *ST*, experimental design and ANOVA analysis were performed using Minitab software. Following the Golden Gate assembly strategy, each gene was individually assembled with its promoter, RBS, and terminator into the pTU1-A ~ D-RFP vectors, generating Level 1 single-gene transcription units. According to experimental design requirements, Level 1 units were sequentially assembled into pTU2-A-RFP to obtain Level 2 polycistronic modules. Assembly procedures followed related literature (Moore et al. 2016; Stukenberg et al. 2021). Plasmid details are provided in Supporting Table S1, primer sequences are listed in Supporting Table S2, and promoter information is provided in Supporting Table S3.

### Multicopy integration of 3’-SL synthesis module

Multicopy chromosomal integration was conducted using a CRISPR-assisted transposase system. crRNAs were designed to target multicopy genomic loci. The system required four plasmids: pDOE13, donor plasmid carrying the *neuB*–*neuC*–*neuA*–*BtST* expression cassette (assembled by Gibson assembly). pTet-tns, expressing transposase proteins TnsA, TnsB, and TnsC. pTetQCas-IS1, carrying the IS1 insertion site, a 32-nt spacer at the target locus, and the auxiliary proteins TniQ, Cas8, Cas7, and Cas6. pCutamp, encoding sgRNAs that remove the other three plasmids after integration. The multicopy integration followed the procedure (Zhang et al. 2020, 2021; Yang et al. 2021). Plasmids and primers are listed in Supporting Table S1 and Supporting Table S2, respectively.

After each transposition cycle, colonies were screened in shake flasks. High-producing strains were subsequently cured of helper plasmids and re-evaluated. To improve plasmid-curing efficiency, induction time in LB medium containing 50 µg mL^−1^ apramycin and 10 mM rhamnose was extended from 3 h to 10 h.

### Fed-batch production in a 5 L bioreactor

Fed-batch fermentation was conducted in a 5 L bioreactor with a 2 L working volume. The fermentation medium contained: 24 g L^−1^ peptone, 12 g L^−1^ yeast extract, 2.0 g L^−1^ NH_4_Cl, 0.24 g L^−1^ MgSO_4_, 6.8 g L^−1^ KH_2_PO_4_, 5 mg L^−1^ FeCl_2_, 10 mg L^−1^ MnCl_2_·4H_2_O, and 5 mg L^−1^ CaCl_2_. Seed cultures were grown by inoculating 1% (v/v) pre-culture into 50 mL LB medium and incubating overnight at 37 °C. The shake-flask culture was transferred to the bioreactor and grown at 37 °C until OD_600_ reached 20. IPTG (0.1 mM) and lactose (10 g L^−1^) were then added, and the temperature was reduced to 25 °C to initiate 3’-SL production.

Dissolved oxygen (DO) was maintained at 15–25% by adjusting agitation (100–1000 rpm), while pH was controlled at ~ 7.0 using 50% ammonia. The initial glucose concentration was 10 g L^−1^. Upon depletion (indicated by rising DO), a feeding solution containing 800 g L^−1^ glucose, 200 g L^−1^ glycerol, and 6.25 g L^−1^ MgSO₄ was supplied continuously. After IPTG induction, lactose was initially added to 10 g L^−1^ and subsequently fed using a DO-stat strategy to maintain a residual concentration of approximately 1–5 g L^−1^. Samples were collected periodically for analysis of OD600, glucose, glycerol, lactose, Neu5Ac, and 3′-SL concentrations.

### Analytical methods

Cell growth was monitored by measuring OD_600_ using a UV-5100 spectrophotometer (Metash, Shanghai). For metabolite analysis, 2 mL fermentation broth was heated at 95°C for 10 min, centrifuged at 12,000 *g* for 10 min, and the supernatant was filtered through a 0.22 µm membrane. Glucose, glycerol, lactose, and 3’-SL were quantified by HPLC using a Sepax HP-Amide column (250 × 4.6 mm, 5 μm) with a mobile phase of acetonitrile: 10 mM ammonium formate (70: 30, v/v). The flow rate was 1.0 mL min^−1^, column temperature 35 °C, and detection at 210 nm. All analyses were performed in triplicate. Data are reported as mean ± standard deviation (SD), and statistical analyses were conducted using Origin 9.0.

## Results and discussion

### Screening of α-2,3-sialyltransferase (ST)

In our previous work, *E. coli* BL21(DE3) was extensively engineered to enhance the Neu5Ac biosynthetic pathway, resulting in strain SLIS026 (with deletions *ΔlacZ*,* ΔnanA*, *ΔnanKE*) (Wu et al. 2022). Subsequently, to further enhance Neu5Ac biosynthesis, the genes encoding N-acetylglucosamine-6-phosphate deacetylase (*NagA*), N-acetylglucosamine-6-phosphate deaminase (*NagB*), and the N-acetylglucosamine-specific phosphotransferase system component (*NagE*) were deleted, yielding strain SLEB01 (Δ*lacZ*, Δ*nanA*, Δ*nanKE*, Δ*nagABE*). Heterologous expression of *neuBC* in SLEB01 enabled Neu5Ac production of 5.5 g L⁻¹ after 44 h of shake-flask fermentation. This strain was subsequently used as the parental chassis for 3’-SL pathway construction and further metabolic engineering. As *E. coli* BL21(DE3) lacks a native 3’-SL biosynthetic pathway, heterologous expression of *neuABC* together with a functional *ST* is required to establish de novo 3’-SL synthesis (Fig. 1). Although the *neuABC* module has been widely studied and is generally not considered a major rate-limiting step, the ST enzyme critically determines 3’-SL production efficiency by catalyzing the formation of the *α*-2,3 glycosidic linkage between CMP-Neu5Ac and lactose. Thus, ST activity and specificity remain key bottlenecks for improving 3’-SL titers.

**Fig. 2:**
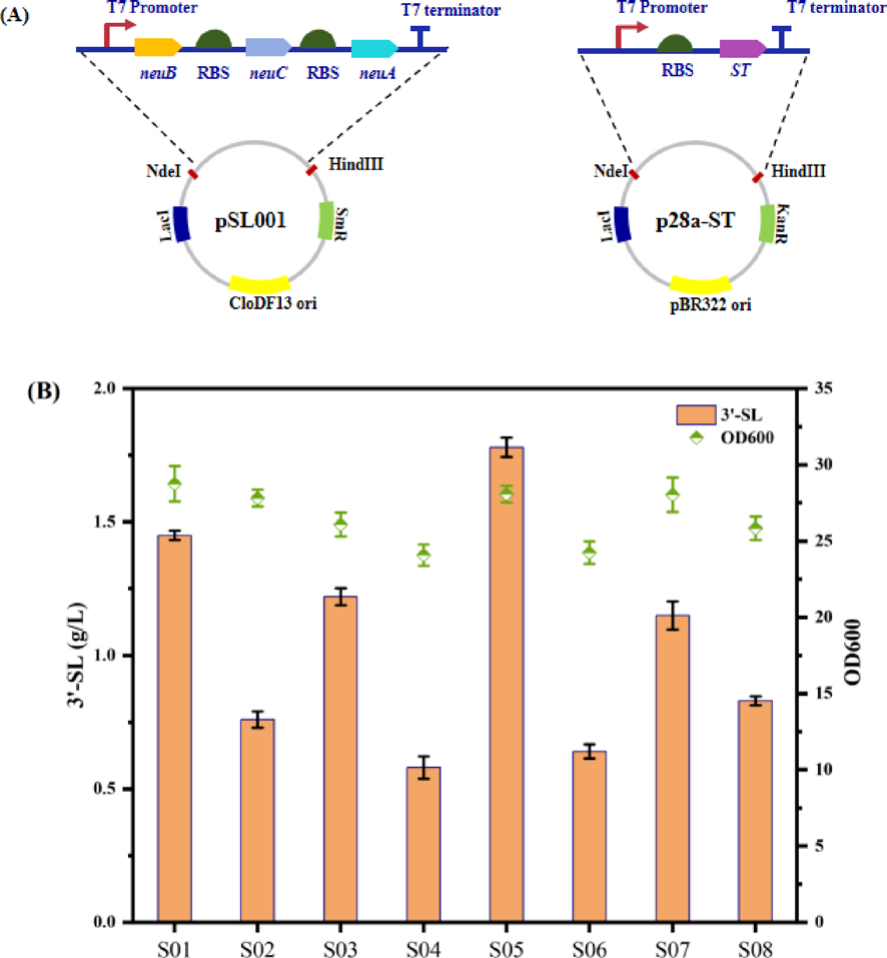
Effect of α-2,3-sialyltransferase (ST) expression from different sources on 3′-SL yield. **A** Plasmid construction procedure. **B** 3′-SL yields and biomass in SLEB01 strains with various ST

To identify an optimal ST candidate, the well-characterized NmST from *Neisseria meningitidis* (AAC44541.1) was used as a reference to search for homologs in the NCBI database. Seven additional ST homologs from phylogenetically diverse species were selected for comparison: PsST (*Photobacterium* sp. JT-ISH-224, BAF92025.1), PmST1 (*Pasteurella multocida*, WP_115173531.1), PmST2 (*Pasteurella multocida*, AAK02272.1), BtST (*Bibersteinia trehalosi*, AGH37861.1), VsST (*Vibrio* sp. JT-FAJ-16, BAF91160.1), NgST (*Neisseria gonorrhoeae*, AAC44539.1), and PpST (*Photobacterium phosphoreum*, BAF63530.1). To evaluate their performance, the four essential genes were divided into two expression modules. The *neuABC* operon was placed on plasmid pCDFDuet-1 (pSL01), whereas each ST candidate was individually expressed from plasmid pET28a (p28a-ST) (Fig. 2A). Co-transformation of the corresponding plasmids into SLEB01 generated eight recombinant strains (S01–S08) for de novo 3’-SL production (Table 1).

Shake-flask fermentation revealed clear differences in 3’-SL production among the ST variants (Fig. 2B). After 48 h of shake-flask fermentation, the BtST-expressing strain (S05) achieved the highest 3’-SL titer (1.78 g L^−1^), followed by the NmST strain (S01) with 1.45 g L^−1^. Sequence alignment showed only 14.87% amino acid sequence identity between BtST and NmST, despite their comparable performance. In contrast, PmST2 (S04) generated the lowest titer (0.57 g L^−1^), whereas its homolog PmST1 (S03) produced 1.22 g L^−1^. These results demonstrate that both the evolutionary origin and sequence divergence of ST markedly influence its in vivo performance, ultimately affecting 3’-SL production.

Previous studies have reported the strong catalytic activity of NmST and PmST, making them the most commonly used STs for constructing high-yield 3’-SL strains (Yu et al. 2005; Fierfort and Samain 2008). BtST, however, has been less explored. Li et al. reported that BtST displayed lower activity than PmST during strain construction, which contrasts with the superior performance observed in our system. This discrepancy may arise from differences in codon optimization strategies, expression levels, or stoichiometric balance between *neuABC* and *ST*, all of which can significantly influence enzyme performance across different host backgrounds (Li et al. 2007). Notably, Talafová et al. reported that BtST exhibits a fourfold higher lactose affinity than *P. multocida* ST, a broader pH tolerance range (6–10), and lower sialidase activity, properties that may favor efficient 3’-SL synthesis (Talafová et al. 2015). Considering these reported advantages and its superior performance in our screening, BtST was selected as the preferred ST candidate for subsequent strain engineering.

### Systematic optimization of the 3’-SL metabolic network via material and energy balancing

**Fig. 3:**
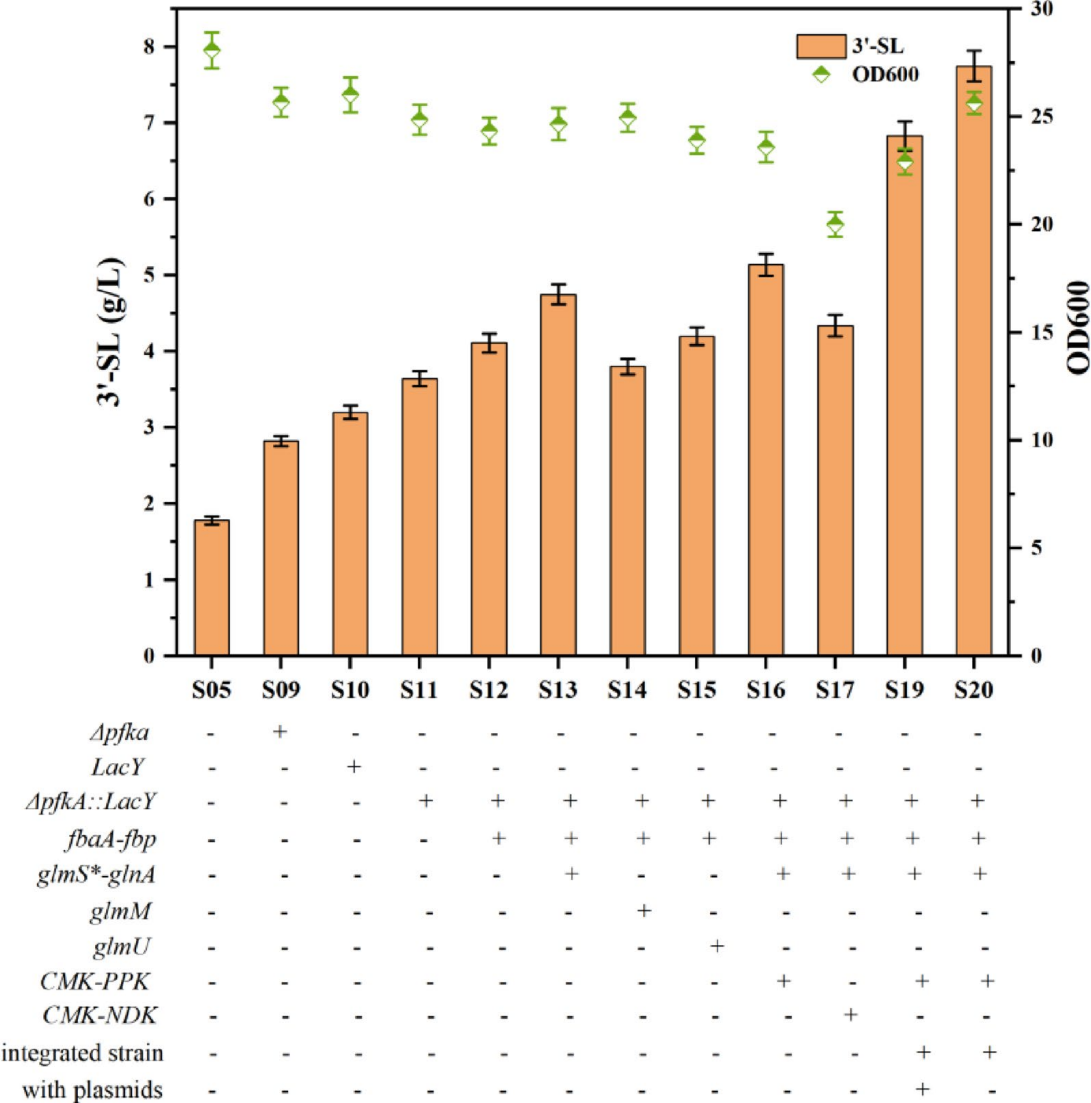
Comparison of engineered strains with deletions or over expression of selected genes “+” indicates the presence of the corresponding genetic modification, whereas “−” indicates its absence

Developing a microbial chassis capable of high-level 3’-SL production requires systematic optimization of the metabolic network to maintain an effective balance between cell growth and product formation. Fructose-6-phosphate (F-6-P) is a key metabolic intermediate that is converted to fructose-1,6-bisphosphate (F-1,6-P) by 6-phosphofructokinase (*pfkA*). This step represents a major regulatory node influencing the partitioning of carbon flux between the Embden-Meyerhof-Parnas (EMP) pathway and the 3’-SL biosynthetic route. Previous studies have shown that *pfkA* deletion reduces carbon flux through the EMP pathway, leading to F-6-P accumulation and consequently increasing precursor availability for target molecules such as 2’-FL and 3’-SL (You et al. 2024; Li et al. 2024a). Following this strategy, *pfkA* was deleted in strain S05 using CRISPR-Cas9, generating strain S09. Compared with the parental strain S05, *pfkA* deletion reduced biomass (OD decreased by 8.5%) but significantly increased the 3’-SL titer by 58.4%. After 48 h of fermentation, the 3’-SL titer of strain S09 reached 2.82 g L⁻¹, indicating enhanced carbon utilization toward the target biosynthetic pathway and a significant enhancement in substrate conversion efficiency (Fig. 3).

Synthesis of 3’-SL requires both glucose metabolism to generate the Neu5Ac precursor and efficient transport of exogenous lactose, the glycosyl acceptor (Lu et al. 2021). Thus, lactose uptake represents another critical factor influencing 3’-SL production. To increase intracellular lactose levels, the endogenous lactose permease gene *lacY* was overexpressed under the tetracycline promoter *Ptet* in strain S05 to create strain S10. After 48 h of shake-flask fermentation supplemented with 5 g L^−1^ lactose, strain S10 produced 3.2 g L^− 1^ 3’-SL, a 79.8% improvement over S05. A slight decrease in growth (7.3% reduction in OD₆₀₀) was observed, which may be attributable to membrane stress associated with *lacY* overexpression. Given the beneficial effects of *pfkA* deletion and *lacY* overexpression on 3’-SL production, these two modifications were combined to construct strain S11. Strain S11 achieved a 3’-SL titer of 3.64 g L^−1^, representing increases of 13.8% and 29.1% compared with strains S10 and S09, respectively. Strains S05-S11 were constructed sequentially, with each strain derived from its immediate parent by introducing a single engineering modification. Among these strains, S11 showed the highest 3’-SL production and was therefore selected for subsequent metabolic engineering.

Considering that fructose-6-phosphate (F-6-P) serves as the entry metabolite for the amino sugar biosynthetic pathway leading to Neu5Ac formation, increasing its intracellular availability was expected to enhance precursor supply for 3′-SL biosynthesis. Therefore, the genes encoding fructose-1,6-bisphosphate aldolase (*fbaA*) and fructose-1,6-bisphosphatase (*fbp*) were overexpressed in strain S11 (Fig. 1). The resulting strain S12 produced 4.11 g L⁻¹ 3’-SL (Fig. 3), representing a 12.9% increase over S11 without significant affecting cell growth. These results suggest that increasing the intracellular F-6-P pool effectively enhanced carbon flux toward 3’-SL biosynthesis.

In metabolic engineering of Neu5Ac-producing strains, the conversion of F-6-P catalyzed by glucosamine-6-phosphate synthase (*GlmS*) is rate-limiting, and this enzyme is inhibited by its product, glucosamine-6-phosphate (GlcN-6-P). A previously reported feedback-resistant mutant, glmS* (E15K/D387V/S450P/E525G), exhibits reduced sensitivity to feedback inhibition by GlcN-6-P (Liu et al. 2025). Because GlmS requires glutamine as an amino donor, overexpression of *glnA* (glutamine synthetase) can increase intracellular glutamine supply and enhance amino sugar biosynthesis pathway (Zhao et al. 2024). Thus, glmS* and *glnA* were co-expressed under *Ptet* in strain S12 to obtain strain S13. Additionally, the downstream genes *glmM* and *glmU*, which catalyze the conversion of GlcN-6-P to UDP-GlcNAc, were overexpressed individually in strain S12, generating strains S14 and S15. Fermentation analysis showed that strain S13 produced 4.75 g L^−1^ of 3’-SL, whereas overexpression of *glmM* or *glmU* alone did not improve titers (Fig. 3). These results indicate that the GlmS-catalyzed amination step is a major bottleneck, and that relieving feedback inhibition while increasing glutamine availability effectively enhances flux toward 3’-SL.

In the final step of 3’-SL synthesis, Neu5Ac must be activated to CMP-Neu5Ac, a reaction requiring CTP (Fig. 1). Li et al. demonstrated that coupling cytidine monophosphate kinase (CMK) with polyphosphate kinase (PPK) enables efficient CTP regeneration during whole-cell catalysis, achieving 52.8 mM 3’-SL within 10 h using exogenous Neu5Ac, lactose, CMP, and polyphosphate (Li et al. , 2021b). To evaluate whether intracellular CTP regeneration could enhance de novo 3’-SL synthesis, we overexpressed the *cmk*-*ppk* or *cmk*-*ndk* modules in strain S13. Strain S16 (*cmk*-*ppk*) increased 3’-SL titer from 4.75 g L^−1^ to 5.14 g L^−1^ (an 8.2% improvement). In contrast, strain S17 (*cmk*-*ndk*) showed reduced production (4.34 g L^−1^, Fig. 3). This difference may be associated with perturbations in cellular nucleotide and energy balance, where ndk overexpression potentially increased ATP turnover, thereby impairing cellular metabolic state and 3’-SL biosynthesis. Furthermore, the modest improvement achieved under shake-flask conditions indicates that cofactor supply is not the primary bottleneck at low production rates. However, in large-scale fermentations, where metabolic flux and product formation rates increase substantially, CTP regeneration may become more critical.

### Adaptive expression of key components in the 3’-SL biosynthetic pathway

#### Identification of key factors influencing 3’-SL pathway efficiency using a Plackett-Burman design

Determining the appropriate expression levels to balance host growth, metabolic burden, and product synthesis remains one of the major challenges in pathway engineering. Excessive expression may impose considerable stress on the host, whereas inadequate expression can cause pathway bottlenecks, leading to inefficient carbon flux distribution or the accumulation of undesirable intermediates. Traditional trial-and-error approaches to pathway optimization require constructing and evaluating numerous gene combinations, making such strategies labor-intensive and limiting in their capacity to explore expression space comprehensively. To address these limitations, we employed a Plackett-Burman experimental design to efficiently screen the key enzymatic steps that most strongly affect 3’-SL biosynthesis. Based on the promoter library constructed by Xu et al. and our laboratory’s prior assessment of promoter strengths, two promoters were selected for combinatorial regulation of four target genes: the strong promoter P2 (+ 1) and the weak promoter P44 (− 1), with promoter strengths listed in Supporting Table S3 (Xu et al. 2016). Application of the Plackett–Burman design reduced the number of required constructs to twelve, significantly improving screening efficiency (Supporting Table 2, Supporting Figure S1).

**Table 2:** Plackett-Burman design to screen main pathway components that determine 3’-SL pathway efficiency with − 1, weak promoter; + 1, strong promoter

Number	neuA	neuB	neuC	BtST	3’-SL (g/L)
1	1	− 1	− 1	− 1	3.09 ± 0.14
2	− 1	− 1	− 1	1	2.65 ± 0.06
3	1	− 1	1	1	4.82 ± 0.10
4	− 1	− 1	− 1	− 1	1.60 ± 0.07
5	1	1	− 1	1	3.38 ± 0.08
6	− 1	− 1	1	1	4.46 ± 0.10
7	− 1	1	1	− 1	2.81 ± 0.06
8	1	1	1	− 1	2.68 ± 0.06
9	1	− 1	1	− 1	3.22 ± 0.07
10	− 1	1	− 1	− 1	2.28 ± 0.11
11	1	1	− 1	1	3.38 ± 0.08
12	− 1	1	1	1	4.31 ± 0.10

All analyses were performed in triplicate. Data are reported as mean ± standard deviation (SD)

**Fig. 4:**
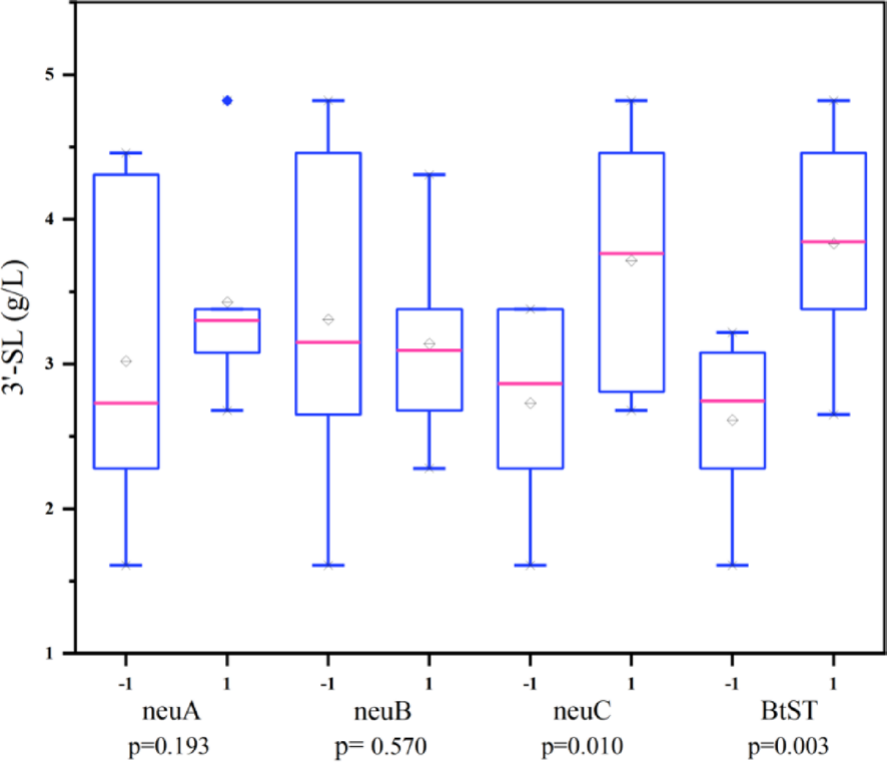
Analysis of variance to determine how significantly each of the pathway components contributes to 3’-SL production. This data point was retrieved from the Plackett−Burman design. Blue box indicates 25 and 75% of the 3’-SL production distribution; × represents 1 and 99% of the 3’-SL production distribution. Pink line indicates the mean value of the data, and diamond point indicates the average of the data

Each construct was individually transformed into the SLEB09 strain (Table 2) and fermented in shake flasks for 48 h. The resulting 3’-SL titers, summarized in Table 2, exhibited pronounced variation across the expression combinations, ranging from 1.6 to 4.82 g L^−1^. One-way analysis of variance (ANOVA) was performed to identify the key factors affecting product formation (Fig. 4). The results revealed that the expression of *neuC* and *BtST* significantly influenced 3’-SL production (*p* < 0.05). Enhancing promoter activity for these two genes consistently improved titers. These observations suggest that *neuC* and *BtST*, representing the initial and final catalytic steps of the heterologous pathway, may exert a synergistic “push–pull” effect: high *neuC* expression enhances conversion of UDP-GlcNAc to ManNAc, while strong BtST activity rapidly channels intermediates into the final product, thereby maintaining balanced metabolic flux and sustaining efficient 3’-SL synthesis. In contrast, variations in *neuA* and *neuB* promoter strength did not significantly affect 3’-SL production (*p* > 0.05), indicating that their expression levels have a relatively minor impact on pathway output. This finding suggests that *neuA* and *neuB* possess sufficiently high basal activities to support 3’-SL biosynthesis across a wide expression range and are therefore less likely to represent rate-limiting steps.

Collectively, the Plackett-Burman design enabled rapid identification of the two key genes (*neuC* and *BtST*) that critically influence 3’-SL pathway performance, effectively narrowing the focus of subsequent optimization efforts. This approach substantially reduced experimental burden, enhanced screening efficiency, and provided a quantitative foundation for rational tuning of gene expression. Overall, the Plackett–Burman design represents a systematic and efficient strategy for evaluating the relative contributions of pathway components, simplifying gene expression optimization, and facilitating metabolic engineering of complex biosynthetic pathways.

#### Optimization of expression levels of key pathway components for 3’-SL synthesis

**Fig. 5:**
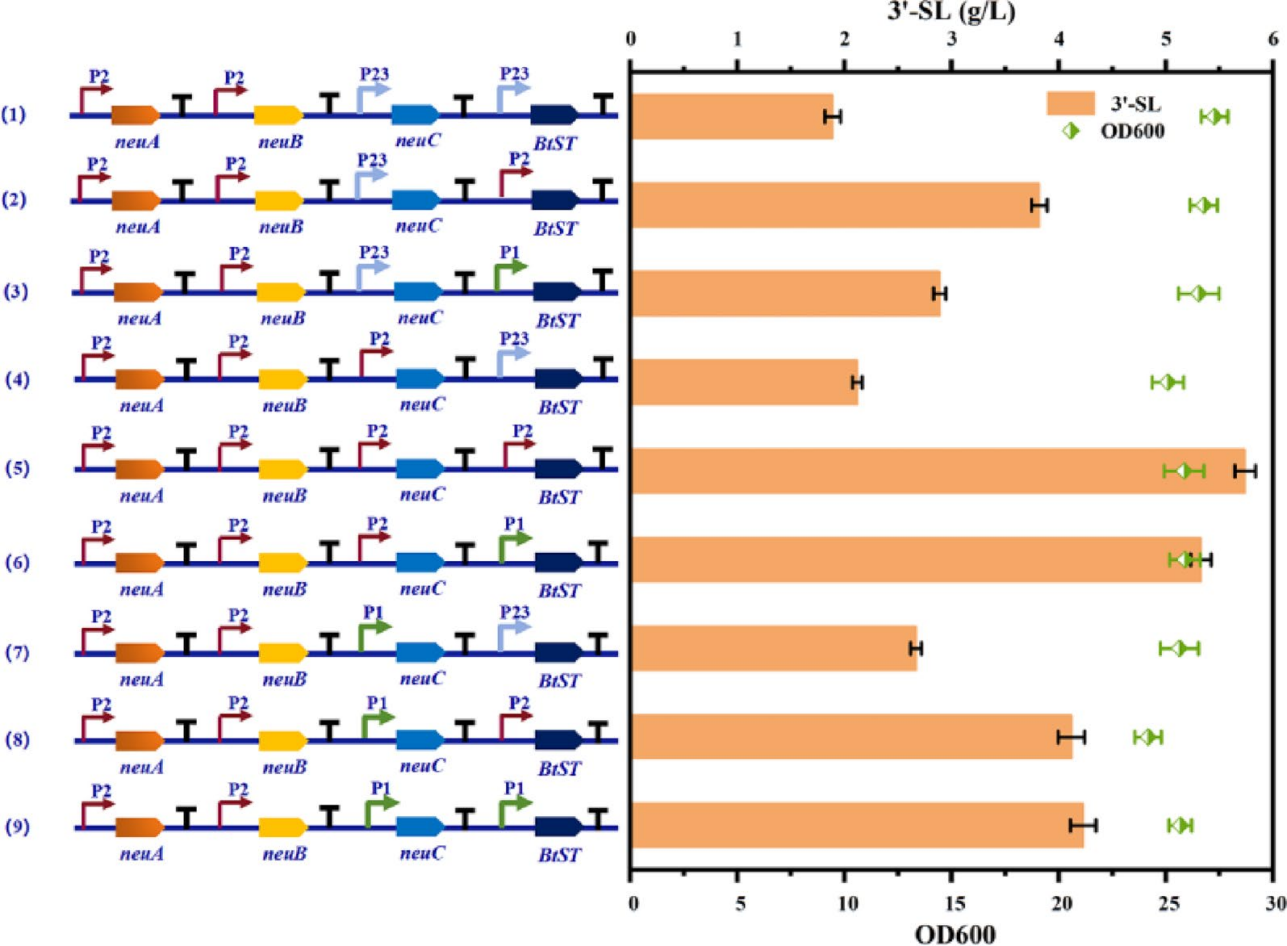
Comparison of engineered strains under different promoter regulation

Based on the results of the Plackett-Burman screening, the expression levels of *neuB* and *neuA* had minimal impact on 3’-SL production. Therefore, promoter P2 was selected to control their expression in subsequent experiments. To fine-tune the expression of *neuC* and *BtST*, a gradient promoter strategy was implemented. Using the P2 promoter as a reference point, the stronger promoter P1 and the weaker promoter P23 were chosen to generate a graded expression series. A two-factor three-level experimental design was applied, yielding nine combinatorial expression constructs (3² = 9). Each construct was introduced into the SLEB09 chassis to generate strains with distinct expression profiles (Fig. 5).

After 48 h of shake-flask fermentation, the strain expressing *neuA*, *neuB*, *neuC*, and *BtST* under promoter P2 exhibited the highest 3’-SL titer of 5.75 g L^−1^ and was designated strain S18 (Fig. 5). Compared with strain S16 (5.14 g L^−1^), which employed a dual-plasmid system, strain S18 using a single-plasmid system with optimized promoter combinations achieved an 11.9% increase in 3’-SL production. Notably, S18 differs from S16 in both promoter configuration and expression architecture, as the key pathway genes were placed under the optimized P2 promoter and consolidated into a single-plasmid system. Therefore, the improved 3’-SL production was was likely driven by the combined effects of promoter optimization and the single-plasmid expression strategy, rather than promoter optimization alone. Moreover, consolidation of the four pathway genes from two plasmids onto a single plasmid simplified pathway construction and provided a convenient platform for the subsequent one-step chromosomal integration.

### Construction and screening of multicopy integrated strains for 3’-SL production

Plasmid-based expression remains the predominant strategy for assembling biosynthetic pathways in microbial hosts. However, plasmids often suffer from genetic instability, uncontrolled copy numbers, and impose a high metabolic burden (Schelch et al. 2023). Chromosomal integration offers a more stable alternative, but iterative multi-gene integration remains challenging. Recent advances in CRISPR-associated transposase systems have enabled simultaneous integration of multiple gene copies through crRNA-guided targeting of multiple genomic loci. Using this method, Zhang et al. achieved up to ten chromosomal copies of glucose dehydrogenase (GDH) in *E. coli* within five days, leading to in a 2.6-fold increase in enzymatic activity compared with plasmid-based expression (Zhang et al. 2020).

Leveraging the optimal *neuA*–*neuB*–*neuC*–*BtST* expression configuration identified earlier, we integrated the multigene cassette into the IS1 insertion site of the SLEB09 chassis, which are present at 30 genomic positions. Transformants were subjected to five rounds of induction to promote multicopy integration and generate strains with varied gene dosages. In each round, 20 colonies were randomly selected for shake-flask fermentation. 3’-SL production increased steadily through the first three induction cycles but plateaued and subsequently declined in rounds four and five (Supporting Figure S2). This suggests that three rounds of integration produced an optimal expression level, whereas additional gene copies imposed excessive metabolic burden, thereby hindering growth and productivity. The strain obtained after three induction rounds was designated strain S19 and yielded 6.83 g L^−1^ of 3′-SL after 48 h (Supporting Figure S2).

Following this, the pCutamp plasmid was introduced into S19 for removal of all auxiliary plasmids. The pTet-tns, pTetQCas-IS1, and pDOE13 plasmids were eliminated via rhamnose induced sgRNA expression, and pCutamp was subsequently removed by sucrose counter-selection. The resulting plasmid-free, multicopy integrated strain was designated S20 (Table 1). To verify chromosomal integration, PCR analysis was conducted for all 30 annotated IS1 loci. Among them, integration of the *neuA*-*neuB*-*neuC*-*BtST* cassette was confirmed at 17 loci (Supporting Figure S3), indicating that strain S20 contains 17 chromosomal integrations generated by the CRISPR-associated transposase system. Strain S20 subsequently produced 7.75 g L⁻¹ of 3’-SL in shake-flask fermentation, representing a 13.5% improvement over S19 (Fig. 3). Its OD_600_ (25.64) was 11.9% higher than that of S19 (22.92), likely due to the absence of leaky expression from helper plasmids, allowing more cellular resources to be allocated to growth and product formation. These findings highlight the advantages of integrating multicopy gene cassettes directly into the genome to enhance genetic stability and production efficiency.

### **Fed-batch production of 3’-SL in a 5**-**L bioreactor**

To evaluate the industrial potential of strain S20, fed-batch fermentation was carried out in a 5-L bioreactor. A mixed glucose–glycerol (4:1) feeding strategy was employed to balance rapid cell growth with efficient carbon utilization during high-cell-density fermentation. Glucose primarily served as the major carbon source for cell growth, whereas glycerol supplementation may have contributed to improved fermentation performance by reducing overflow metabolism and supporting production during the late stage of fermentation.

**Fig. 6:**
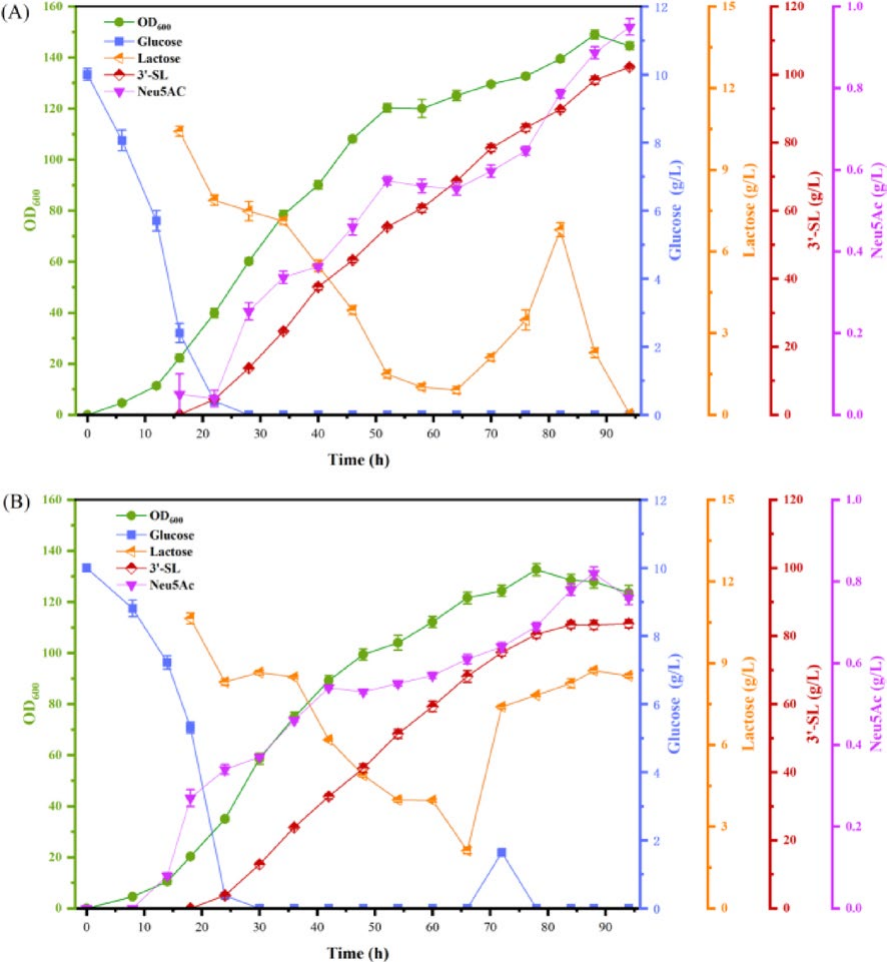
Production of 3’-SL by strains S20 (A) and S18 (B) in a 5-L bioreactor

During fed-batch fermentation of strain S20 (Fig. 6A), biomass increased steadily and reached a maximum OD_600_ of 148.9 at 88 h, indicating robust strain performance and suggesting that 3’-SL accumulation did not impose substantial growth inhibition. After 94 h of cultivation, the OD_600_ began to decline, likely due to excessively high OD_600_ levels and insufficient dissolved oxygen, which impaired cellular viability. Under the DO-stat feeding strategy, the residual glucose concentration was maintained at nearly 0 g L^−1^ after approximately 30 h, indicating that the glucose feeding rate was lower than or closely matched the cellular glucose consumption rate, thereby preventing glucose accumulation and minimizing overflow metabolism throughout the fermentation. Glycerol feeding was also controlled via the DO-stat strategy, resulting in negligible residual glycerol throughout the process, generally below the detection limit. Therefore, glycerol concentration data are not shown in Fig. 6A.

3’-SL accumulation began at approximately 22 h and entered a rapid production phase, ultimately reaching 102.18 g L⁻¹ at 94 h, corresponding to a 13-fold increase over shake-flask fermentation, with an average productivity of 1.09 g L^−1^ h^−1^ (Fig. 6A). Throughout the fermentation, residual Neu5Ac remained at a consistently low concentration, suggesting that Neu5Ac did not accumulate as a major intermediate. This observation may reflect either limited Neu5Ac biosynthesis or its rapid conversion to CMP-Neu5Ac. Further analyses, such as intracellular metabolite quantification and metabolic flux analysis, will be needed to clarify the underlying mechanism. Lactose was maintained at approximately 1–5 g L⁻¹ for most of the fermentation via DO-stat feeding. During the rapid production phase (16–64 h), lactose was consumed rapidly, resulting in a gradual decrease in residual lactose. After 64 h, the lactose consumption rate declined, leading to a transient increase in residual lactose until lactose feeding was terminated at 82 h. The remaining lactose was subsequently depleted. Overall, approximately 210 g lactose was consumed, corresponding to a molar conversion efficiency of 88.1%. In addition, strain S20 consumed a total of 1,120 g of glucose and 280 g of glycerol, corresponding to substrate requirements of 3.27 g glucose and 0.82 g glycerol per gram of 3’-SL produced.

To assess the impact of chromosomal integration on fermentation performance, strain S20 was compared with the plasmid-based strain S18 under identical 5-L fed-batch fermentation conditions. As shown in Fig. 6B, strain S18 reached a maximum OD_600_ of 123.65 and produced 83.64 g L^−1^ of 3’-SL after 94 h of cultivation. A total of 181.4 g lactose was consumed, corresponding to a lactose molar conversion efficiency of 83.5%. In contrast, strain S20 achieved a 20.4% higher maximum biomass (OD_600_ of 148.9), a 22.2% higher final 3’-SL titer (102.18 g L^−1^), and a modest improvement in lactose conversion efficiency (88.1%). Notably, the fermentation profiles of the two strains differed markedly during the late production stage. From 78 to 94 h, 3’-SL production by strain S18 increased only marginally, whereas strain S20 continued to accumulate product throughout this period. This difference may be associated with reduced plasmid stability or increased metabolic burden during prolonged cultivation. The sustained productivity of strain S20 contributed to its higher final titer and improved substrate utilization efficiency. These results demonstrate the superior robustness of chromosomal integration during long-term fermentation. To the best of our knowledge, the 3’-SL titer achieved by strain S20 represents the highest reported for microbial de novo 3’-SL production to date, highlighting its potential for large-scale industrial production.

## Conclusions

In this study, we developed a high-performance microbial cell factory for de novo 3’-SL production by integrating metabolic engineering, pathway expression optimization, and CRISPR-assisted multicopy chromosomal integration. This strategy generated the plasmid-free and antibiotic marker-free strain S20 with enhanced genetic stability for high-density fermentation. As summarized in Table 3, strain S20 showed superior fermentation performance compared with previously reported microbial de novo 3′-SL production systems. Compared with the plasmid-based strain S18, S20 exhibited more sustained production during prolonged fermentation, highlighting the benefit of multicopy chromosomal integration for constructing robust production strains.

**Table 3:** Comparison of research advances in microbial fermentation for 3’-SL Production

strain	strategies	titer (g/L)	productivity (g/L/h)	Lactose conversion rate (mol 3′-SL/mol)	References
*E. coli* BL21(DE3)	Δ*lacZ*, Δ*nanA*, Δ*nanK*, recA::Ptac-*Vs16*; harboring 3 plasmids: pET-*neuBCA2A*, pCDF-*Vs16* and pRSF-glmMUS*	31.4 (5 L bioreactor)	0.82		Zhang et al. 2022
*E. coli* BL21(DE3)	Δ*lacZ*, Δ*nanA*, Δ*pfkA*, Δ*nanK*, Δ*manA*; harboring 3 plasmids: pET-*neuBCA*-*glnA*, pCDF-*glmMUS* and pRSF-*ST*-*lacY*	44.2 (3 L bioreactor)	0.53	0.55	Li et al. ,[Bibr CR9][Bibr CR9]
*E. Coli* BL21star (DE3)	Δ*lacZ*, Δ*nanA*, Δ*nanK*, Δ*nanE*, Δ*nanT*, Δ*nagB*, Δ*pfkB*, P*pfkA*::pL19 PT7-*lacY* PT7-*scrY*; chromosomally integrated *neuBCA*-*nST*-*glmSMU*-*cmk*	56.8 g/L (5 L bioreactor)	0.19	0.55	Lv et al. 2024
*E. coli* BL21(DE3)	Δ*lacZ*, Δ*nanA*, Δ*nanK*; harboring 2 plasmids: pET-*BCA* and pCD-406NST*	29.54 (5 L bioreactor)	0.52	0.62	Lin et al. 2025
JM109(DE3) + *S. cerevisiae*	JM109(DE3) harboring two plasmids: pET-*ce_3*-*nanA* and pCDF-*nst3*-*neuA*; coupled with *Saccharomyces cerevisiae* for CTP regeneration	78.03 (5 L bioreactor)	3.25	0.3079 mol/mol GlcNAc	Zhang et al. 2025
*E. coli* BL21(DE3)	Δ*lacZ*, Δ*nanA*, Δ*nanT*; chromosomally integrated Nst*; harboring 2 plasmids: pET-*neuBCA* and pCDF-NST*	32.1 (5 L bioreactor)	0.68	N/A	Zhang et al. 2024
*E. coli* BL21(DE3)	*ΔLacZ*,* ΔnanA*,* ΔnanKE*,* ΔnagABE*,* ΔpfkA::LacY*,* ΔaslA::fbaA-fbp*,* ΔgltB::glmS-glnA*,* ΔmelB::CMK-PPK;* chromosomally integrated *neuA*-*neuB*-*neuC*-*BtST* gene cassette	102.18 (5 L bioreactor)	1.09	0.88	This study

Despite the high production level achieved in this study, additional improvements remain possible. Future efforts may focus on engineering BtST to further enhance its catalytic efficiency, optimizing glucose uptake by modifying the PtsG transport system to increase phosphoenolpyruvate (PEP) availability, and further refining fermentation strategies to improve lactose conversion efficiency. Moreover, quantitative analyses of intracellular nucleotide pools and metabolic fluxes would provide a deeper understanding of pathway limitations and guide the next round of strain optimization.

Overall, this work establishes an effective strategy for constructing genetically stable microbial cell factories for 3’-SL production and provides a versatile engineering framework for the biosynthesis of other HMOs and high value bioproducts.

## Supplementary Information

Below is the link to the electronic supplementary material.

Supplementary Material 1

## Data Availability

All data generated or analysed during this study are included in this published article [and its supplementary information files].

## Declarations

### Supplementary Information

Summary of the plasmids, primers and promoters used in this study (Table S1, Table S2, Table S3); Genetic configuration of the constructed pathways that conforms to the Plackett-Burman design (Figure S1); Screening of genomic multicopy integration strains (Figure S2); PCR verification of the copy number and insertion site of *neuA*-*neuB*-*neuC*-*ST* in strain S20 (Figure S3).
