# Supplementary material for: Systematic pathway engineering of Escherichia coli for enhanced 3’-sialyllactose production
Source: Bioresour Bioprocess. 2026 Aug 10;13(1):112. doi: 10.1186/s40643-026-01110-8 (PMC13457516; doi:10.1186/s40643-026-01110-8)
Supplement: Supplementary file 1 [file 40643_2026_1110_MOESM1_ESM.docx]

**Supplementary Information**

Systematic Pathway Engineering of *Escherichia coli* for Enhanced 3’-Sialyllactose Production

Yan Wu^a^, Taoling Min^a^, Zhenfeng Ma^a^, Shu Wang^a^, Chuanmin Sun^a^, Hanzhi Zhang^a^, Chenxi Huang^b^, Xudong Qu^a,c*^, Zhenhua Tian^a*^

^a^Abiochem Biotechnology (Group) Co., Ltd., Shanghai 200241, China

^b^Synaura Biotechnology (Shanghai) Co., Ltd., Shanghai 200120, China

^c^State Key Laboratory of Microbial Metabolism and School of Life Sciences and Biotechnology, Shanghai Jiao Tong University, Shanghai, 200240, China

^*^ Corresponding authors.

Email addresses: quxd@whu.edu.cn and [tianzhenhua@abiochem.com](mailto:tianzhenhua@abiochem.com).

**Figure S1.** Genetic configuration of the constructed pathways that conforms to the Plackett-Burman design (Table 1). All of the genes are organized in monocistronic form to ensure context-independent gene expression. +1, strong promoter (P2); −1, weak promoter (P44); T, terminator.

**Figure S2**. Screening of genomic multi-copy integration strains.

**Figure S3.** PCR verification of the copy number and insertion site of *neuA-neuB-neuC-ST* in strain S20.

**Table S1**. Plasmids used in this study.

| Plasmids | Characteristics | source |
| --- | --- | --- |
| pSL01 | SpecR, pCDFDuet1 plasmids carrying *neuA-neuB-neuC* gene cassette | This tudy |
| pET28a-ST | KanR，carrying different *ST* gene | This study |
| pCas | KanR, repA101(Ts), cas9, ParaB-Red, PrhaB-sgRNA-pMB1, sacB | Lab stock |
| pTargetF | SmR, J23119 promoter, gRNA scaffold | Lab stock |
| pSL02-lacZ | pTargetF, lacZ-targeting spacer | This study |
| pSL03-pfkA | pTargetF, pfkA-targeting spacer | This study |
| pSL04-melB1 | pTargetF, melB1-targeting spacer (knock in *lacY*) | This study |
| pSL05-gltB | pTargetF, gltB-targeting spacer (knock in *glmS*-glnA/glmM/glmU*) | This study |
| pSL06-aslA | pTargetF, aslA-targeting spacer (knock in *fbaA-fbp*) | This study |
| P07-melB2 | pTargetF, melB2-targeting spacer (knock in *CMK-PPK/CMK-NDK*) | This study |
| pTU1-A-RFP | Used for assembling the promoter, RBS or purification tag, A gene or ORF, and terminator in Level 0.AmpR | Lab stock |
| pTU1-B-RFP | Used for assembling the promoter, RBS or purification tag, B gene or ORF, and terminator in Level 0.AmpR | Lab stock |
| pTU1-C-RFP | Used for assembling the promoter, RBS or purification tag, C gene or ORF, and terminator in Level 0.AmpR | Lab stock |
| pTU1-D-RFP | Used for assembling the promoter, RBS or purification tag, D gene or ORF, and terminator in Level 0.AmpR | Lab stock |
| pTU2-A-RFP | Used for assembling the four transcription units A to D in Level 1.cmR | Lab stock |
| pSL08 | pTU1-A-RFP backbone carrying P2-*neuA*-T7T gene cassette | This study |
| pSL09 | pTU1-A-RFP backbone carrying P44-*neuA*-T7T gene cassette | This study |
| pSL10 | pTU1-B-RFP backbone carrying P2-*neuB*-T7T gene cassette | This study |
| pSL11 | pTU1-B-RFP backbone carrying P44-*neuB*-T7T gene cassette | This study |
| pSL12 | pTU1-C-RFP backbone carrying P2-*neuC*-T7T gene cassette | This study |
| pSL13 | pTU1-C-RFP backbone carrying P44-*neuC*-T7T gene cassette | This study |
| pSL14 | pTU1-D-RFP backbone carrying P2-*BtST*-T7T gene cassette | This study |
| pSL15 | pTU1-D-RFP backbone carrying P44-*BtST*-T7T gene cassette | This study |
| pSL16 | pTU1-C-RFP backbone carrying P23-*neuC*-T7T gene cassette | This study |
| pSL17 | pTU1-D-RFP backbone carrying P23-*BtST*-T7T gene cassette | This study |
| pSL18 | pTU1-C-RFP backbone carrying P1-*neuC*-T7T gene cassette | This study |
| pSL19 | pTU1-D-RFP backbone carrying P1-*BtST*-T7T gene cassette | This study |
| pSL20 | pTU2-A-RFP backbone carrying P2-*CSS*-T7T, P2-*neuB*-T7T, P2-*neuC*-T7T and P2-*BtST*-T7T gene cassette | This study |
| pTet-tns | KanR, carrying three types of transposase proteins (*TnsA*, *TnsB*, *TnsC*) | Lab stock |
| pTetQCas-IS1 | SmR, TetR, tniQ, cas678, IS1 locus (30) | Lab stock |
| pRE57 | AmpR, lacI, RE, and LE | Lab stock |
| pCutamp | ApmR, PrhaB-sgRNA-AmpR promoter, for the elimination of  pTet-*tns*, pTetQCas-IS1, and pRE57 plasmids | Lab stock |
| pDOE13 | pRE57 backbone, carrying P2-*CSS*-T7T, P2-*neuB*-T7T, P2-*neuC*-T7T and P2-*ST*-T7T gene cassette | This study |

**Table S2**. Primers used in this study.

| Primer | Sequence (5'→3') |
| --- | --- |
| *neuB*-F | tttaactttaataaggagatataccatgaaagaaatcaaaatccagaaca |
| *neuB*-R | aattttcttcatggtatatctccttttaggcgaaatcttcataagacagc |
| *neuC*-F | aaggagatataccatgaagaaaattctgtttatcaccg |
| *neuC*-R | CTGTTTTTCCATggtatatctccttttatttaatatctttgaacactttc |
| *CSS*-F | aaggagatataccATGGAAAAACAGAACATCGCGGTTA |
| *CSS*-R | tgctcagcggtggcagcagcctaggttaAGATTCTTTGTGGTTCAGGATG |
| pSL01-F | cctaggctgctgccaccgctg |
| pSL01-R | ggtatatctccttattaaagttaaa |
| sgRNA-*LacZ*-F | TTACGCCAGCTGGCGAAAGGgttttagagctagaaatag |
| sgRNA-*LacZ*-R | AGCCCGGACTCGGTAATGGCGCGCATgaattcaaaaaaagcaccgactcg |
| L-*LacZ*-F | ATGCGCGCCATTACCGAGTCC |
| L-*LacZ*-R | AGGGCGATCGGTGCGGGCCTCGACGGCCAGTGAATCCGTA |
| R-*LacZ*-F | TACGGATTCACTGGCCGTCGAGGCCCGCACCGATCGCCCT |
| R-*LacZ*-R | ataacagggtaatagatctaagcttAGTAGGGTAGTGATGAGAGGCTT |
| pTarget-Hind III-F | aagcttagatctattaccctgttat |
| *LacZ*-R | CCTTTCGCCAGCTGGCGTAAactagtattatacctaggac |
| sgRNA-*pfkA*-F | gtttctgacatgatcaaccggttttagagctagaaatag |
| sgRNA-*pfkA*-R | tcatatcccatgcgtaacgcgctatgaattcaaaaaaagcaccgactcg |
| L-*pfkA*-F | atagcgcgttacgcatgggat |
| L-*pfkA*-R | gtttctttctcgatgaaatgcgctgtcagcgcagaacgaacaac |
| R-*pfkA*-F | gttgttcgttctgcgctgacagcgcatttcatcgagaaagaaac |
| R-*pfkA*-R | ataacagggtaatagatctaagcttatgttaaccctacgccccact |
| pTarget-Hind III-F | aagcttagatctattaccctgttat |
| *pfkA*-R | cggttgatcatgtcagaaacactagtattatacctaggac |
| *melB*-F | CAAAGCATTACATTAAGACGgttttagagctagaaatag |
| *melB*-R | CGTCTTAATGTAATGCTTTGactagtattatacctaggac |
| tet-*lacY*-F | cggggttgttcgttctgcgctgacaTAAGGCCAGATGATTAATTCCTAAT |
| *lacY*-R | cggtttctttctcgatgaaatgcgcTTATTAAGCGACTTCATTCACCTGA |
| R-pSL05-F | gcgcatttcatcgagaaagaaacc |
| L-pSL05-R | tgtcagcgcagaacgaacaacc |
| *gltB*-F | TTGCTCAATGCGTGGCAGAGgttttagagctagaaatag |
| *gltB*-R | CTCTGCCACGCATTGAGCAAactagtattatacctaggac |
| L-*melB*-F | ggctttgctatctattatttctcat |
| L-*melB*-R | cgtcaggctttcacatcactca |
| tet-*melB*-F | tgagtgatgtgaaagcctgacgTAAGGCCAGATGATTAATTCCTAAT |
| tet-R | TTGTATATCTCCTTCTTAAAGTTAA |
| *lacY*-tet-F | TTAACTTTAAGAAGGAGATATACAAATGTACTATTTAAAAAACACAAACT |
| *LacY*-tet-R | atccggtctacaaaatttgcaaatcTTATTAAGCGACTTCATTCACCTGACG |
| R-melB-F | gatttgcaaattttgtagaccggat |
| R-melB-R | atgacccgcacactcaagcc |
| *cmk*-F | TTAACTTTAAGAAGGAGATATACAAatgacggcaattgccccggtt |
| *cmk*-R | tgctcagcggtggcagcagcctaggttatgcgagagccaatttctggc |
| T7T-*cmk*-F | cctaggctgctgccaccgctg |
| T7T-*cmk*-R | cgaaacctgataaacatatcgcatccaaaaaacccctcaagacccg |
| pt5-*ppk*-F | cgggtcttgaggggttttttggatgcgatatgtttatcaggtttcg |
| pt5-*ppk*-R | cgatgtatagcttttcctgacccatatgtatatctccttgtgtatttata |
| *ppK*-F | tataaatacacaaggagatatacatatgggtcaggaaaagctatacatcg |
| *ppK*-R | atccggtctacaaaatttgcaaatcttattcaggttgttcgagtgatttg |
| *ndk*-F | TTAACTTTAAGAAGGAGATATACAAatggctattgaacgtactttttcca |
| *ndk*-R | tgctcagcggtggcagcagcctaggttaacgggtgcgcgggcacac |
| L-*gltB*-F | attcgcaaagcagatccagca |
| L-tet-R | AAAATTAGGAATTAATCATCTGGCCacgcgcgcctcgcattcgccc |
| tet-*gltB*-F | GGCCAGATGATTAATTCCTAATTTT |
| tet-*glms*-R | TTGTATATCTCCTTCTTAAAGTTAA |
| *glmS*-tet-F | TTAACTTTAAGAAGGAGATATACAAatgtgcggtatcgttggtgcta |
| *glmS*-tet-R | AAAATTAGGAATTAATCATCTGGCCTTAttattccacggtcacggatttcg |
| *glnA*-tet-F | ttaactttaagaaggagatatacaaatgtccgctgaacacgtactga |
| *glnA*-R | CAAGACCCGTTTAGAGGCCCCAAGGGGTTATGCTAGttagacgctgtagtacagctcaa |
| R-*gltB*-F | TGGGGCCTCTAAACGGGTCTTGAGGGGTTTTTTGggttaaaccgaagtccagtgatgta |
| R-*gltB*-R | atgctgacgaacggttcgtcg |
| *glmM*-F | TTAACTTTAAGAAGGAGATATACAAatgagtaatcgtaaatatttcggta |
| *glmM*-R | CAAGACCCGTTTAGAGGCCCCAAGGGGTTATGCTAGttaaacggcttttactgcatcgg |
| *glmU*-F | TTAACTTTAAGAAGGAGATATACAAatgttgaataatgctatgagcgtag |
| *glmU*-R | CAAGACCCGTTTAGAGGCCCCAAGGGGTTATGCTAGtcactttttctttaccggacgac |
| P2-pSL07-F | ggccgcttctagaacgtctcaatctCTATGGATAATCAGATCACGAAATTAATA |
| RBS-pSL07-R | atgtatatctccttcttaaagttaa |
| *neuA*-F | ttaactttaagaaggagatatacatATGGAAAAACAGAACATCGCGG |
| *neuA*-R | GCTTTGTTAGCAGCCGGATCtcgaGGATCCttaAGATTCTTTGTGGTTCAGGATG |
| T7T-*neuA*-F | GGATCCtcgaGATCCGGCTGCTAACAAAGC |
| T7T-pSL07-R | gaggaagcctgcagcgtctctggcaaacaCAAAAAACCCCTCAAGACCCG |
| pSL07-F | tgccagagacgctgcaggcttcctc |
| pSL07-R | agattgagacgttctagaagcgg |
| P44-*neuA*-F | cggccgcttctagaacgtctcaatctCTATGGATCGAGATCGATCTCGATCC |
| P2-pSL09-F | ggccgcttctagaacgtctcatgccCTATGGATAATCAGATCACGAAATTAATA |
| RBS-pSL09-R | tgttctggattttgatttctttcatatgtatatctccttcttaaagttaa |
| *neuB*-F | atgaaagaaatcaaaatccagaaca |
| *neuB*-R | GTTAGCAGCCGGATCtcgaGGATCCttaggcgaaatcttcataagacagc |
| T7T-*neuB*-F | GGATCCtcgaGATCCGGCTGCTAACAAAGC |
| T7T-*neuB*-R | ctgcagcgtctctccggaacaCAAAAAACCCCTCAAGACCCG |
| pSL09-F | CGGGTCTTGAGGGGTTTTTTGtgttccggagagacgctgcag |
| pSL09-R | ggcatgagacgttctagaagcggcc |
| P44-*neuB*-F | ggccgcttctagaacgtctcatgccCTATGGATCGAGATCGATCTCGATCC |
| P2-pSL011-F | ggccgcttctagaacgtctcaccggCTATGGATAATCAGATCACGAAATTAATA |
| RBS-pSL011-R | atgtatatctccttcttaaagttaa |
| *neuC*-F | ttaactttaagaaggagatatacatatgaagaaaattctgtttatcaccg |
| *neuC*-R | GGCTTTGTTAGCAGCCGGATCtcgaGGATCCttatttaatatctttgaacactttc |
| T7T-*neuC*-F | GGATCCtcgaGATCCGGCTGCTAACAAAGCC |
| T7T-*neuC*-R | aagcctgcagcgtctctcttcaacaCAAAAAACCCCTCAAGACCCGT |
| pSL011-F | tgttgaagagagacgctgcaggctt |
| pSL011-R | ccggtgagacgttctagaagcggcc |
| P44-*neuC*-F | ggccgcttctagaacgtctcaccggCTATGGATCGAGATCGATCTCGATCC |
| P23-*neuC*-F | ggccgcttctagaacgtctcaccggCTATGGATGCCCCGATCCTCAAATTA |
| P2-pSL013-F | ggccgcttctagaaCGTCTCAGAAGCTATGGATAATCAGATCACGAAATTAATA |
| RBS-pSL013-R | atgtatatctccttcttaaagttaa |
| *BtST*-F | ttaactttaagaaggagatatacatATGGAGTTCTGCAAAATGGCGA |
| *BtST*-R | GCTTTGTTAGCAGCCGGATCtcgaGGATCCTTATTCTTTGGTCAGCAGCTGCA |
| T7T-*BtST*-F | GGATCCtcgaGATCCGGCTGCTAACAAAGC |
| T7T-*BtST*-R | gaagcctgcagCGTCTCTCTAAaacaCAAAAAACCCCTCAAGACCCG |
| pSL013-F | tgttTTAGAGAGACGctgcaggcttc |
| pSL013-R | ATAGCTTCTGAGACGttctagaagcggcc |
| P44-*BtST*-F | ggccgcttctagaaCGTCTCAGAAGCTATGGATCGAGATCGATCTCGATCC |
| P23-*BtST*-F | ggccgcttctagaaCGTCTCAGAAGCTATGGATGCCCCGATCCTCAAATTA |
| P1-*neuC*-F | ggccgcttctagaacgtctcaccggCTATGGTTTGCCCCGATCGGTAAA |
| P1-*BtST*-F | ggccgcttctagaaCGTCTCAGAAGCTATGGTTTGCCCCGATCGGTAAAT |
| pSL020-F | attgataattatcacacccagaattcgcggccgcttcta |
| pSL020-R | accgtattaccgcctttgag |
| LacI-pSL020-F | ctcaaaggcggtaatacggttcactgcccgctttccagt |
| Lacl-LE145-R | tcatcaccgaaacgcgcgaggacaccatcgaatggcgca |
| pRE57-F | ctcgcgcgtttcggtgatga |
| pRE57-R | tgggtgtgataattatcaatttatgggtg |

**Table S3.** Promoters used in the experiments.

| Promoter | Relative Strength | Sequence (5'→3') |
| --- | --- | --- |
| P1 | 1.73 | GGTTTGCCCCGATCGGTAAATTAATACAGCTCACTATAGGAATCGTGTACGGGGGACTATTCC |
| P2 | 1.0 | GGATAATCAGATCACGAAATTAATACCACTCACTATAGGAATAGTGCCCGGGTAACCATTCC |
| P23 | 0.70 | GGATGCCCCGATCCTCAAATTAATACCACTCACTATAGGAATGGTAGCCGGGGGACGATTCC |
| P44 | 0.01 | GGATCGAGATCGATCTCGATCCCGCGAAATTAATACGACTCACTATAGGGGAATTGTGAGCGGATAACAATTCC |
| T7 | 0.12 | TAATACGACT CACTATAGGG GAATTGTGAG CGGATAACAA TTCC |
